# Intact and Defective HIV Provirus Changes During Antiretroviral Therapy in People Treated During Acute or Chronic HIV Infection or as HIV Controllers

**DOI:** 10.1093/ofid/ofaf568

**Published:** 2025-09-16

**Authors:** Rajesh T Gandhi, Joshua C Cyktor, Ronald J Bosch, Hanna Mar, Gregory M Laird, Albine Martin, Sharon A Riddler, Paul E Sax, Jonathan Z Li, Deborah K McMahon, John W Mellors, Joseph J Eron

**Affiliations:** Department of Medicine, Massachusetts General Hospital and Harvard Medical School, Boston, Massachusetts, USA; Department of Medicine, University of Pittsburgh School of Medicine, Pittsburgh, USA; Department of Biostatistics, Harvard TH Chan School of Public Health, Boston, USA; Department of Biostatistics, Harvard TH Chan School of Public Health, Boston, USA; Accelevir, Inc, Baltimore, USA; Accelevir, Inc, Baltimore, USA; Department of Medicine, University of Pittsburgh School of Medicine, Pittsburgh, USA; Department of Medicine, Brigham and Women's Hospital and Harvard Medical School, Boston, USA; Department of Medicine, Brigham and Women's Hospital and Harvard Medical School, Boston, USA; Department of Medicine, University of Pittsburgh School of Medicine, Pittsburgh, USA; Department of Medicine, University of Pittsburgh School of Medicine, Pittsburgh, USA; Department of Medicine, University of North Carolina, Chapel Hill, North Carolina, USA

**Keywords:** antiretroviral therapy, HIV controllers, HIV persistence, HIV, provirus, HIV reservoirs

## Abstract

**Background:**

Intact proviral DNA (IPD) is a measure of the replication-competent HIV reservoir. Little is known about how IPD levels compare in people with HIV (PWH) who initiate antiretroviral therapy (ART) during acute HIV infection (AHI), chronic infection (CHI) or as HIV controllers (CON).

**Methods:**

Participants with sustained plasma HIV RNA < 50 copies/mL on ART had longitudinal measurements of intact, defective and total proviral DNA in blood samples.

**Results:**

Twenty-nine participants were evaluated: 14 CHI, 7 AHI and 8 CON. PWH-CON had lower IPD than PWH-AHI or PWH-CHI during ART. PWH-CON also had low intact and total provirus levels before initiating ART. During years 2–5 of ART, IPD decay half-life was 1.0 years in PWH-AHI, 1.6 years in PWH-CHI and 3.2 years in PWH-CON (*P* = .01 for PWH-CON vs PWH-AHI). Defective provirus levels did not decrease in PWH-AHI and PWH-CHI.

**Conclusions:**

During the initial years of ART, PWH treated during acute and chronic infection have decay in intact but not defective proviruses. PWH controllers have low intact and total provirus levels before and during ART, suggesting interactions between host and virus shape the proviral landscape. Variable proviral decay patterns in these populations provide insight into approaches to achieve ART-free HIV remission.

A subset of replication-competent intact HIV-1 proviruses leads to HIV rebound when antiretroviral therapy (ART) is stopped. Measurement of intact HIV proviruses provides an estimate of the size of the persistent HIV reservoir that must be eliminated or reduced to make progress toward ART-free HIV remission [1, 2]. Several studies have shown that there is a decline in intact HIV proviruses, but not total HIV proviruses, after initiation of ART [3–5]. The decline in intact HIV proviruses is most rapid during the first five to seven years of ART but then slows substantially during the second decade of ART [3, 6, 7]. In some instances, there is an increase in IPD levels during long-term ART, most likely because of proliferation of infected CD4^+^ T cell clones [6, 7].

Most data on intact HIV proviral decay after initiation of ART, however, come from studies of people who initiated treatment during chronic HIV infection. Less is known about intact proviral decay in people who started ART during acute HIV infection or as HIV controllers (people with HIV who are not receiving ART and have HIV RNA levels < 1000 copies/mL). During the first 6 months of ART, people treated during acute infection may have more rapid decay of intact proviruses than people treated during chronic infection; however, whether this more rapid decay continues is not certain [8]. People who initiate ART as HIV controllers may have a lower absolute number of proviruses than non-controllers because pre-ART HIV RNA (which is lower in HIV controllers) is associated with total HIV DNA once on ART [9]. Whether intact proviral DNA decay differs between HIV controllers and non-controllers is not known.

Here, we compare intact, defective and total HIV proviral DNA levels and decay during the initial years of therapy in people with HIV who initiated ART during acute infection, chronic infection and as HIV controllers.

## METHODS

### Study Population

People with HIV (PWH) who had sustained and documented plasma HIV RNA < 50 copies/mL on ART and without known ART interruption were enrolled into a longitudinal cohort (ACTG A5321). PWH with chronic infection (PWH-CHI) initiated ART in ACTG trials for treatment-naïve persons and had follow-up while continuing to receive ART (ACTG studies A5001 and A5321) [9 ]. PWH treated during acute HIV infection (PWH-AHI) had initiated ART during acute infection, as defined by the following testing patterns: (1) enzyme or chemiluminescence immunoassay (E/CIA) nonreactive and HIV-1 RNA or p24 antigen-positive or (2) 4th or later generation FDA-approved E/CIA reactive and a negative or HIV-1 indeterminate Geenius or Western blot. PWH who were treated as HIV controllers (PWH-CON) had pre-ART HIV RNA levels consistently <1000 copies/mL prior to ART initiation; most participants had been enrolled in a previous ACTG study, A5308 [10]. Participants were selected based on the availability of stored cells during initial years of ART for proviral DNA testing; PWH-CHI participants were previously described [6]. Participants in all three groups had suppressed plasma HIV RNA levels by commercial assays starting at week 48 of ART and at all subsequent time points (plasma HIV RNA levels < 50 copies/mL; no confirmed HIV RNA results ≥ 50 copies/mL; isolated HIV RNA results 50 to 200 copies/mL were allowed) and no reported ART interruptions.

### Patient Consent Statement

The institutional review boards at the authors’ institutions approved the study. All participants provided written informed consent for their participation in the study (only adult participants were enrolled).

### Virologic Assays

We performed the following measurements on CD4^+^ T-cells isolated from cryopreserved peripheral blood mononuclear cells (PBMC): intact proviral DNA, 5′ defective or 3′ defective/hypermutated proviral DNA, and total proviral DNA (sum of intact and defective/hypermutated proviruses), assayed by IPDA^®^ at Accelevir Diagnostics as previously reported [1]. Samples were additionally mobilized for two PWH-CHI and one PWH-AHI, but intact proviral DNA was not analyzable due to amplification or detection failure which is determined by a lack of positive droplets for either of the two amplicons in the IPDA. In the analyzed participants, a result could have zero intact proviruses (no droplets double-positive for both amplicons), but there would be single-positive droplets detected for both amplicons. The number of cell equivalents assayed was determined for each sample, and proviral DNA measurements were normalized per million CD4^+^ T-cells. The limit of detection was dependent on the number of CD4^+^ T-cells available for the assay at each timepoint. We also measured low-level plasma residual viremia by automated multireplicate single copy HIV-1 RNA assay (SCA), which is a multireplicate adaptation of the Aptima HIV-1 Quant assay on the Hologic Panther platform [11]. Plasma (5 mL) was centrifuged in a Thermo Scientific Sorvall Legend X1 centrifuge accommodating the Hologic Panther Specimen Aliquot Tubes prior to being loaded on the Hologic Panther instrument and run according to manufacturer recommendations. The SCA lower limit of 0.49 copies/mL is based on the reported result of <0.49 copies/mL for the case where all 9 replicates in the assay are negative.

### Statistical Analysis

Estimation of the decay in intact proviral DNA during the initial years of ART was performed using linear mixed effects models (random intercept) applied to log_10_-transformed intact proviral DNA frequencies. These models were developed to incorporate left-censored (below assay limit) results [12, 13]. For timepoints with no intact provirus detected, these were analyzed as being left-censored at 1/(number of CD4^+^ T-cells assayed). From the model-estimated slopes over time and confidence intervals (CIs), corresponding half-life estimates and CIs were calculated. Based on prior findings of an inflection point and plateauing subsequent to the initial decline of intact DNA frequencies, analyses of decay were restricted to before year 5 of ART [3, 6]. Cross-sectional associations between intact proviral frequencies and SCA were assessed using Spearman correlations, analyzing SCA values < 0.49 copies/mL as the lowest rank and analyzing intact proviral frequencies below the highest left-censored value as the lowest rank.

## RESULTS

### Study Population

Twenty-nine participants who had samples from 1–2 years after ART initiation to 4–6 years after ART initiation were evaluated: 14 PWH-CHI, 7 PWH-AHI and 8 PWH-CON. Participant demographics and pre-ART HIV RNA and CD4^+^ T-cell count results are shown in Table 1. All eight PWH-CON had pre-ART HIV RNA values < 1000 copies/mL; two of the eight PWH-CON had pre-ART HIV RNA < 50 copies/mL (elite controllers).

**Table 1.** Participant Characteristics for Individuals Who Initiated ART During Chronic Infection (PWH-CHI), During Acute Infection (PWH-AHI) or as a Controller (PWH-CON), and Summaries of Plasma Viremia on ART Measured by Single Copy Assay (SCA)

		Chronic (N = 14)	Acute (N = 7)	Controller (N = 8)	Total (N = 29)
Sex	Male	9 (64%)	6 (86%)	5 (63%)	20 (69%)
	Female	5 (36%)	1 (14%)	3 (37%)	9 (31%)
Gender	Male	9 (69%)	6 (86%)	5 (63%)	20 (71%)
	Female	4 (31%)	1 (14%)	3 (37%)	8 (29%)
Pre-ART age (y)	Median	44	42	53	45
	Q1–Q3	33–48	32–59	41–56	35–53
Pre-ART plasma HIV RNA (log_10_ cps/mL)^a^	Median	4.2	6.0	2.4	4.2
	Q1–Q3	4.0–5.0	4.2–6.2	1.9–2.7	2.7–5.3
Below 1000 cps/mL	<1000	2 (14%)	0 (0%)	8 (100%)	10 (34%)
Below 50 cps/mL	<50	0 (0%)	0 (0%)	2 (25%)	2 (7%)
Pre-ART CD4^+^ T-cell count (cells/mm³)	Median	377	485	817	474
	Q1–Q3	216–524	332–710	448–1011	324–754
ART year 2 HIV RNA by SCA (cps/mL)	Median	0.86	0.80	<0.49	<0.49
	<0.49	6 (43%)	3 (43%)	7 (88%)	16 (55%)
ART year 4–6 HIV RNA by SCA (cps/mL)^b^	Median	0.58	<0.49	<0.49	<0.49
	<0.49	7 (50%)	5 (71%)	6 (86%)	18 (64%)

^a^Pre-ART HIV RNA for PWH-CHI was the geometric mean of the last 2 pre-ART HIV RNA results prior to ART initiation in their ACTG ART-naive clinical trial. Pre-ART HIV RNA for the other participants was the last pre-ART HIV RNA result.

^b^Missing SCA in one PWH-CON at year 4–6. Due to sample availability, SCA from either year 4 or 6 for each participant is summarized.

### Intact and Total Proviral DNA Levels Are Lower in HIV Controllers

**Figure 1.**
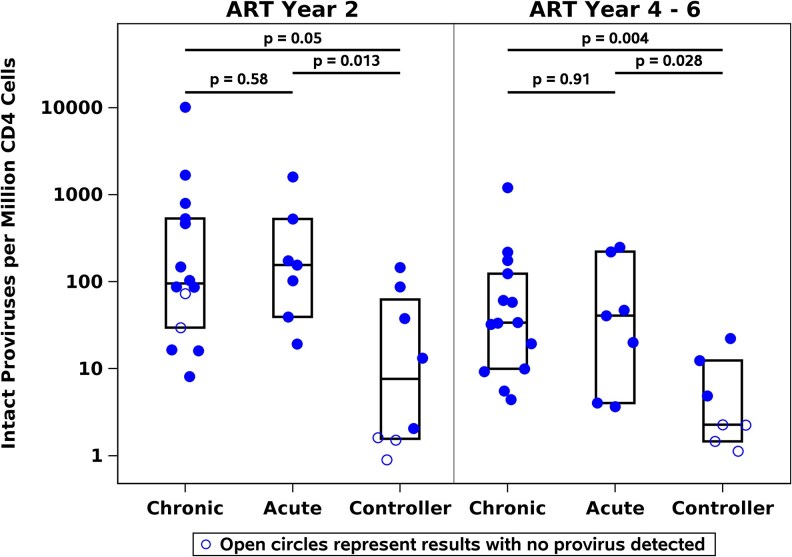
Intact proviral DNA levels are lower in ART-treated HIV controllers (PWH-CON) than in PWH treated during chronic or acute HIV infection (PWH-CHI, PWH-AHI). Statistical comparisons based on the Wilcoxon rank-sum test, analyzing results with no detected intact proviral DNA as the lowest rank.

At year 2 after ART initiation, PWH-CON had significantly lower IPD levels than PWH-CHI (median 8 vs 95 DNA copies/million CD4^+^ T-cells, *P* = .05) and PWH-AHI (median 8 vs 155 copies/million CD4^+^ T-cells, *P* = .013) (Figure 1). At year 4–6, PWH-CON continued to have lower IPD than PWH-CHI (median < 2.3 vs 34 copies/million CD4^+^ T-cells, *P* = .004) and PWH-AHI (median < 2.3 vs 41 copies/million CD4^+^ T-cells, *P* = .028) (Figure 1). Total proviruses were also lower in PWH-CON than in PWH-CHI and PWH-AHI: at year 4–6 of ART, median 25, 252 and 415 copies/million CD4^+^ T-cells, respectively (*P* = .002 and *P* = .015, respectively; *P* = .85 comparing PWH-CHI vs PWH-AHI; Supplementary Figure 1).

### Impact of ART on Intact Proviral DNA in HIV Controllers

**Figure 2.**
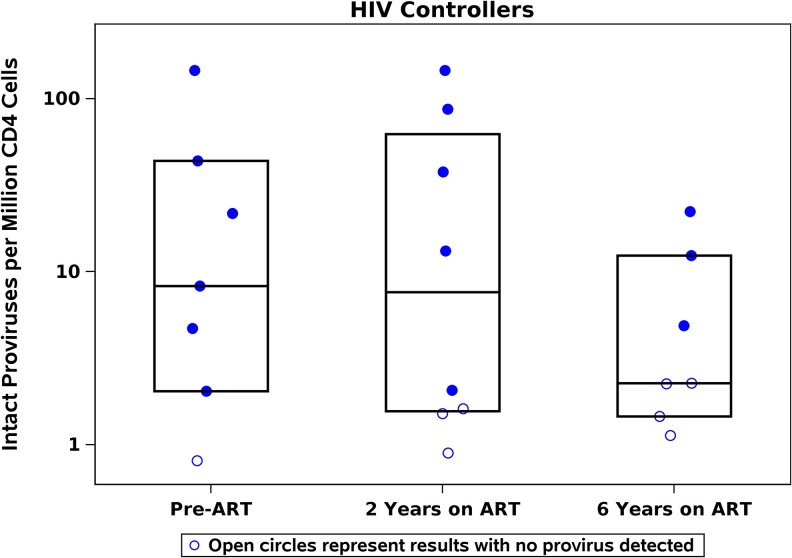
Minimal change after ART initiation in the frequency of intact proviruses in HIV controllers (PWH-CON).

To assess the impact of ART on IPD in PWH-CON, levels from a pre-ART time point were compared to on-ART time points (Figure 2) (such samples were not available for PWH-AHI and PWH-CHI). PWH-CON had low levels of intact, defective and total DNA proviruses before ART (median of 8, 28 and 37 copies/million CD4^+^ T-cells, respectively). PWH-CON had little absolute change in IPD levels between pre-ART time point (8 copies/million CD4^+^ T-cells) and on-ART time points (year 2: 8 copies/million CD4^+^ T-cells; year 6: < 2.3 intact proviral copies/million CD4^+^ T-cells). However, the proportion of participants with intact proviruses below limits of detection increased over time (pre-ART, 1 out of 7 participants (14%); year 2, 3 out of 8 participants (38%); year 6, 4 out of 7 participants (57%)).

### Decay of Intact, Defective and Total Proviruses During Years 2 to 5 of ART

**Figure 3.**
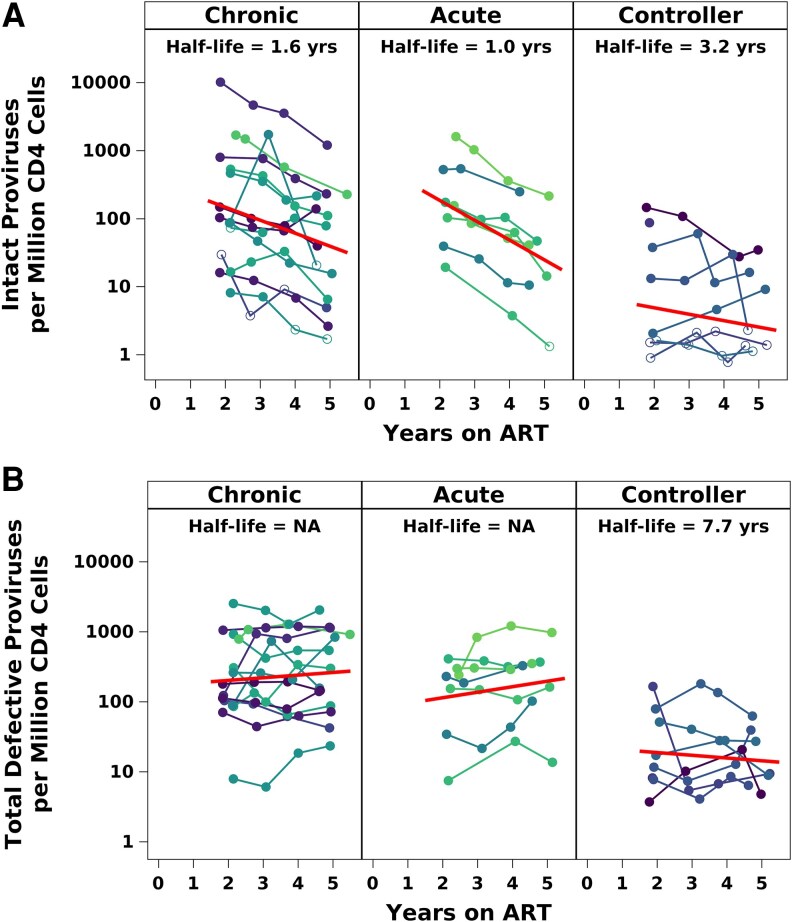
Decay of HIV proviruses (intact, panel *A*; defective, panel *B*) during years 2–5 of ART. Each line represents values over time from an individual participant. Half-life estimates and corresponding group-specific curves (shown in red; see straight lines) were obtained from linear mixed effects model developed to incorporate left-censored (below assay limit) results.

We assessed longitudinal levels of intact, defective and total proviral DNA during years 2–5 of ART which were available for all groups. As displayed in Figure 3*A*, individual participants generally showed continual declines in intact proviral frequencies during years 2–5 of suppressive ART. The decay over time seen on the log_10_-transformed scale supports the linear modeling approach, which corresponds to estimating the half-life of intact proviral decay separately for each group. The number of CD4^+^ T-cells analyzed for an IPDA result was generally more than 350 000 cells (Supplementary Table 1). Decay of intact proviral DNA was faster for PWH-AHI (estimated half-life 1.0 years, 95% CI: 0.7–1.8 years) than for PWH-CHI (1.6 years, 95% CI: 1.2–2.2 years), but the difference was not statistically significant (*P* = .13). Intact proviral decay was slower for PWH-CON and significantly slower in comparison to PWH-AHI (half-life 3.2 years, 95% CI: 1.7–27 years; *P* = .15 and *P* = .01 vs PWH-CHI and PWH-AHI, respectively).

Because many CON participants had non-detected intact proviral DNA, a sensitivity analysis excluding the four participants in the controller group with the lowest pre-ART levels was also done, which gave similar results (PWH-CON half-life 3.0 years over years 2 to 5, n = 4). A sensitivity analysis was also performed of all intact DNA levels over years 1 to 5 of ART since the PWH-CON and PWH-CHI groups had available samples and intact DNA measurements also at ART year 1; estimated half-lives were 3.3 and 1.4 years for PWH-CON and PWH-CHI, respectively (*P* = .02 comparing PWH-CON and PWH-CHI). These half-life estimates were similar to results over years 2 to 5 of ART (3.2 and 1.6 years for PWH-CON and PWH-CHI groups, respectively; Figure 3*A*).

There was no evidence of decay in defective provirus in the PWH-CHI and PWH-AHI groups over years 2–5 (Figure 3*B*), while PWH-CON showed decay in defective proviruses with half-life of 7.7 years (95% CI: 2.9-infinity years). For all three groups, the confidence intervals for change over years 2–5 included zero (no change), consistent with no evidence for decay in defective provirus frequencies.

Changes in the intact percentage (intact/total proviruses) paralleled changes in intact frequencies, with greater decreases for PWH-AHI and PWH-CHI than for PWH-CON. Between year 2 and years 4–6 of ART, the selective decline in intact proviruses in PWH-AHI and PWH-CHI corresponded to declines in intact percentages for PWH-AHI from median 54% to 12%, and for PWH-CHI from 44% to 13% as compared to no apparent changes for PWH-CON, from median 22% to <28%.

### Low-Level Residual Viremia

Plasma HIV viremia was measured by single copy assay (SCA) at years 2 and years 4–6 of ART in PWH-CHI, PWH-AHI and PWH-CON (Table 1). A high proportion of participants had HIV RNA below the limits of quantification (0.49 copies/mL); this proportion appeared to be highest in PWH-CON (7 out of 8, 88% at year 2 of ART) but the numbers of participants in each group were too small to make any statistical comparisons. Notably, at both year 2 and years 4–6 of ART, plasma HIV RNA by SCA was positively correlated with intact proviral DNA frequencies (r = 0.67, *P* < .001 and r = 0.58, *P* = .001, respectively; Supplementary Figures) combining the CHI, AHI and CON participants. Plasma HIV RNA by SCA also correlated at year 2 and years 4–6 with defective proviral frequencies (r = 0.48, *P* = .008 and r = 0.51, *P* = .006) and with total proviruses (r = 0.59, *P* < .001 and r = 0.52, *P* = .004).

In addition, PWH-CHI who had detectable SCA values ≥0.49 copies/mL at year 2 of ART typically continued to have detectable SCA values at year 4 and 6 of ART; conversely, PWH-CHI who had SCA values below assay limit (<0.49 copies/mL) usually continued to have SCA values below that threshold at year 4 and 6 of ART (Supplementary Tables).

## DISCUSSION

During years 2–5 of ART, people with HIV (PWH) treated during acute and chronic infection have significant decays in intact but not defective proviruses. By contrast, treated PWH controllers have low intact and total proviral DNA levels before and during ART, suggesting pre-ART interactions between host and virus shape the HIV proviral landscape that then persists during treatment.

Previous work has shown selective decline in intact proviral DNA during ART in people treated during chronic HIV infection [3, 4, 5]. The current study finds that people treated during acute HIV infection also have selective and rapid decline of intact proviral DNA during years 2–5 after ART initiation. Intact proviral DNA decay in people treated during acute infection (half-life, 1.0 year) is slightly faster than people treated during chronic infection (half-life, 1.6 years), although with the current sample size the difference in half-lives was not statistically significant. If the observation is confirmed in larger studies, more rapid intact proviral decay in people treated during acute infection than during chronic infection may reflect more effective immunity in the former group leading to more rapid immune-mediated clearance of infected cells.

Notably, the relatively rapid decay of intact proviruses during the initial years of ART in people treated during chronic HIV infection does not continue during longer durations of treatment. Previous work from our group and others has shown an inflection point starting at around five years of ART after which intact proviral DNA decay slows markedly and sometimes even reverses [3, 6, 7]. When analyses examine IPDA changes involving timepoints well beyond ART year 5, half-life estimates are substantially longer than our findings over years 2–5 of ART [3, 5, 7, 14]. The more rapid initial intact proviral DNA decay following ART initiation in persons treated during chronic infection may represent elimination of transcriptionally active proviruses that are intermittently expressed. The slowing of subsequent intact proviral DNA decay as ART continues may be related to persistence of transcriptionally silent proviruses. Alternatively, multiple studies show proliferation of infected CD4 T cell clones during long-term ART, which may result in persistence or even expansion of the intact proviral HIV DNA reservoir [7].

We also characterized intact proviral DNA changes in people who are HIV controllers. We find that people who are HIV controllers have low levels of intact and total HIV proviruses, which is consistent with a previous study reporting that HIV elite controllers have lower frequencies of total and intact proviruses than non-controllers [15]. However, the previous study evaluated elite controllers who maintained HIV RNA below the limit of clinical assays and were not receiving ART and compared their proviral DNA levels to non-controllers who were receiving ART. In addition, only a single time point was evaluated. By contrast, in the current study, we find that HIV controllers (most of whom were not elite controllers) who were receiving ART continued to have lower total and intact proviral DNA levels with significantly slower decay than PWH treated during acute or chronic infection and that difference persisted over several years.

There are several possible explanations for why HIV controllers have lower levels of intact and total proviruses and significantly slower decay than PWH treated during acute or chronic infection. First, before ART is initiated, HIV controllers have lower plasma HIV RNA levels than non-controllers. Because higher pre-ART HIV RNA is associated with higher on-ART proviral DNA levels, lower HIV RNA levels in HIV controllers would be expected to be accompanied by lower HIV DNA proviral levels [9]. Second, the pre-ART CD4^+^ T-cell count in people treated as HIV controllers (median of 817/mm^3^) was higher than people treated during acute and chronic infection (median of 485 and 377/mm^3^, respectively). Previous studies have found that higher pre-ART (nadir) CD4 cell count correlates with lower on-ART intact proviral DNA [3]. Finally, HIV controllers may have had many years during which the host immune system has eliminated intact proviruses present in transcriptionally active regions of the human genome, leaving behind proviruses that are more transcriptionally quiescent [16]. The remaining transcriptionally silent proviruses are not expressed and, therefore, may persist.

In addition to evaluating longitudinal changes in proviral DNA, we also examined levels of residual low-level viremia in people treated during chronic HIV infection using a new automated multireplicate single copy assay [11]. These SCA measurements positively correlated with frequencies of intact as well as defective and total proviruses. In prior work [2], we had not seen a significant correlation between SCA and IPDA levels which might have been due to substantial heterogeneity in the duration on ART at sampling in the prior study (4 to 17 years) and to higher sensitivity of the multireplicate SCA assay [11] used in the current study compared to single-replicate, manual SCA used in the prior study [2]. We found that participants who had detectable RNA values at year two of ART typically continued to have detectable SCA values at year 4 and 6 of ART; conversely, those who had SCA values below assay limit (<0.49 copies/mL) usually continued to have SCA values below that threshold at subsequent time points. This finding of consistent levels of residual viremia over time suggests that the automated single copy assay may be useful in clinical trials to assess the impact of interventions on low-level viremia and the HIV reservoir.

Our study has several limitations, including the small number of participants in each group. Participants diagnosed and treated during acute infection are difficult to find. Furthermore, HIV controllers are uncommon, and investigators in the ACTG A5308 study (from which the controllers in this study were enrolled) found that many potentially eligible participants declined to go on ART. Because we wanted to evaluate changes over time, we also needed participants who had provided longitudinal specimens. Due to limited sample availability and our interest in longer term changes, we did not evaluate IPDA during the first year of ART; a sensitivity analysis showed that IPDA half-lives were similar over years 2–5 as compared to years 1–5 of ART for the two groups with IPDA at year 1. In contrast to other reports [17, 18], IPDA levels on ART were similar for the PWH-CHI and PWH-AHI participants in our study; this could be due to relatively lower pre-ART HIV RNA levels and correspondingly higher pre-ART CD4^+^ T-cell count levels in our PWH-CHI group (median 377 cells/mm^3^), since lower nadir (pre-ART) CD4 count is associated with higher on-ART IPDA levels [3]. It is important to highlight that the two-primer IPDA used in our study is an upper bound on the replication-competent HIV reservoir, but correlates with frequencies based on viral outgrowth measurements [1, 2, 7, 19]. In addition, the IPDA might not detect not all intact viruses due to sequence diversity resulting in primer mismatch. Limited sample availability for our study participants did not allow for parallel evaluation of other reservoir measurements. However, for both acute-treated and chronic-treated individuals on ART in another report, relative changes over time in IPDA levels closely matched corresponding relative changes in viral outgrowth frequencies for the majority of participants, including multiple participants evaluated during the initial six years of on-ART viral suppression [19]. Future studies should evaluate larger numbers of participants treated during acute infection or as HIV controllers while ensuring similar timepoints on ART whenever assessing differences. Additional work should also compare the integration sites and genomic locations of proviruses in people treated during acute and chronic infection and as HIV controllers.

Our study has several important implications. If participants treated during acute HIV infection have more rapid decay in intact proviruses after ART initiation than people who receive ART later, the former group may be more likely to achieve HIV remission following interventions designed to accelerate intact proviral elimination. However, it is likely that people treated during acute infection will have slowing in intact proviral DNA decay during longer-term ART, leading to similar challenges in achieving HIV remission as are seen in people treated during chronic infection. The finding that people who are HIV controllers have low proviral DNA levels suggests that we need a deeper understanding of how the host immune system molds the HIV proviral landscape before ART is initiated; those insights may yield clues into how to reduce or alter the HIV reservoir to the extent needed to achieve HIV remission.

## Supplementary Material

ofaf568_Supplementary_Data

## Supplementary Data

Supplementary materials are available at *Open Forum Infectious Diseases* online. Consisting of data provided by the authors to benefit the reader, the posted materials are not copyedited and are the sole responsibility of the authors, so questions or comments should be addressed to the corresponding author.
