# Supplementary material for: Intact and Defective HIV Provirus Changes During Antiretroviral Therapy in People Treated During Acute or Chronic HIV Infection or as HIV Controllers
Source: Open Forum Infect Dis. 2025 Sep 16;12(10):ofaf568. doi: 10.1093/ofid/ofaf568 (PMC12481157; doi:10.1093/ofid/ofaf568)
Supplement: ofaf568_Supplementary_Data [file ofaf568_supplementary_data.pdf]

Supplemental Figure 1

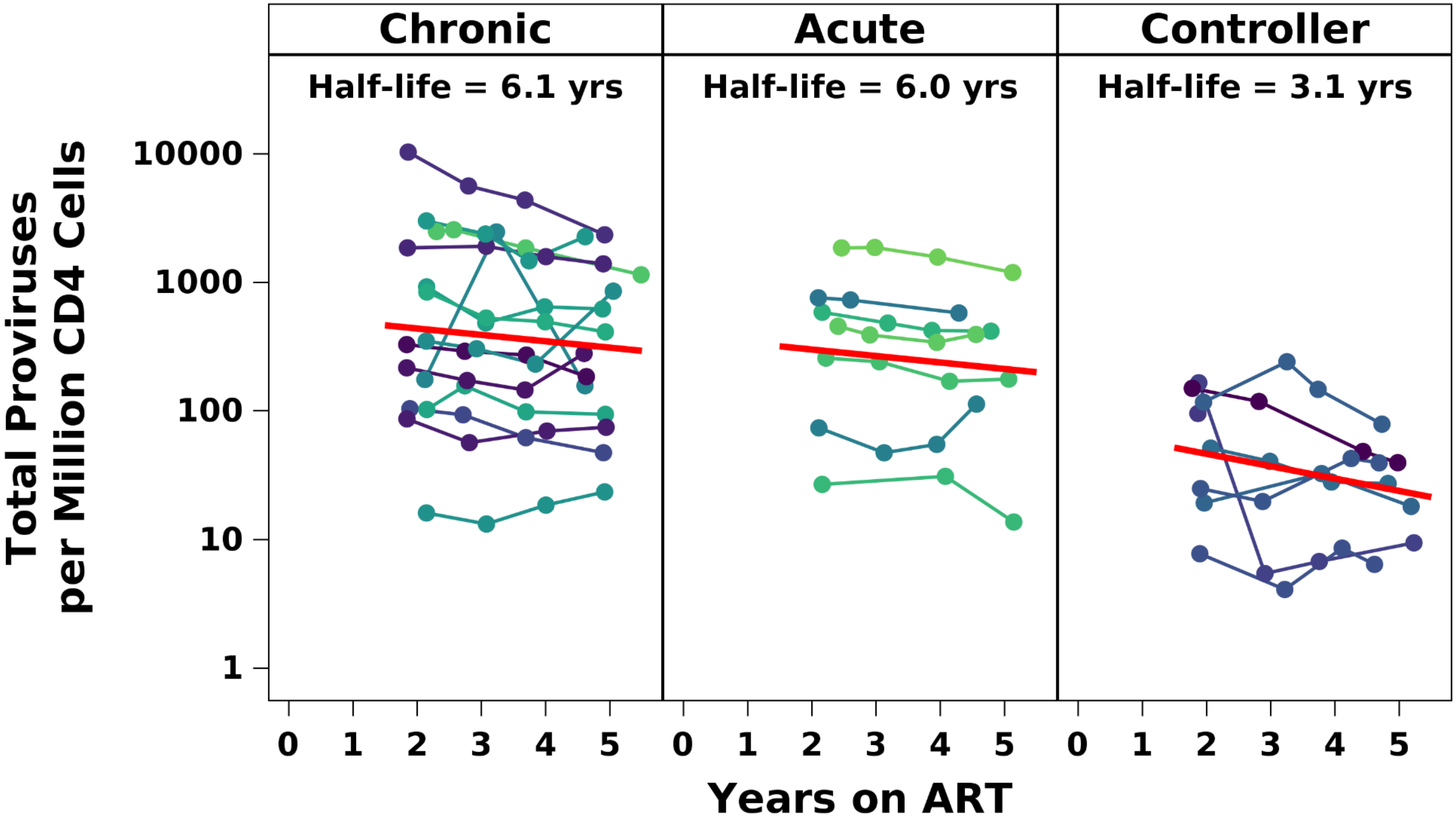

Supplemental Figure 1:

Decay of total proviruses (intact + defective) during ART years 2 - 5. Half-life estimates from linear mixed effects model.

Supplemental Table 1: Number of CD4 Cell Equivalents, Overall and by IPDA Detected/Not Detected, During ART Years 2 - 5

		**Detected (N=88)**	**Not Detected (N=21)**	**Total (N=109)**
Number of Cell Equivalents	Median	603,307	620,767	603,679
	Q1 - Q3	348,067 - 895,553	428,685 - 736,589	351,908 - 850,646
	10% - 90%	249,219 - 1,209,307	48,524 - 1,036,054	240,822 - 1,166,308

Created by: /home/actg/A5321/final/IPDA_GROUPS1_2_3_LEUK/publication/manuscript/programs/supptab_celleq.sas on June 24, 2025

Supplemental Figure 2: SCA vs IPDA at ART year 2

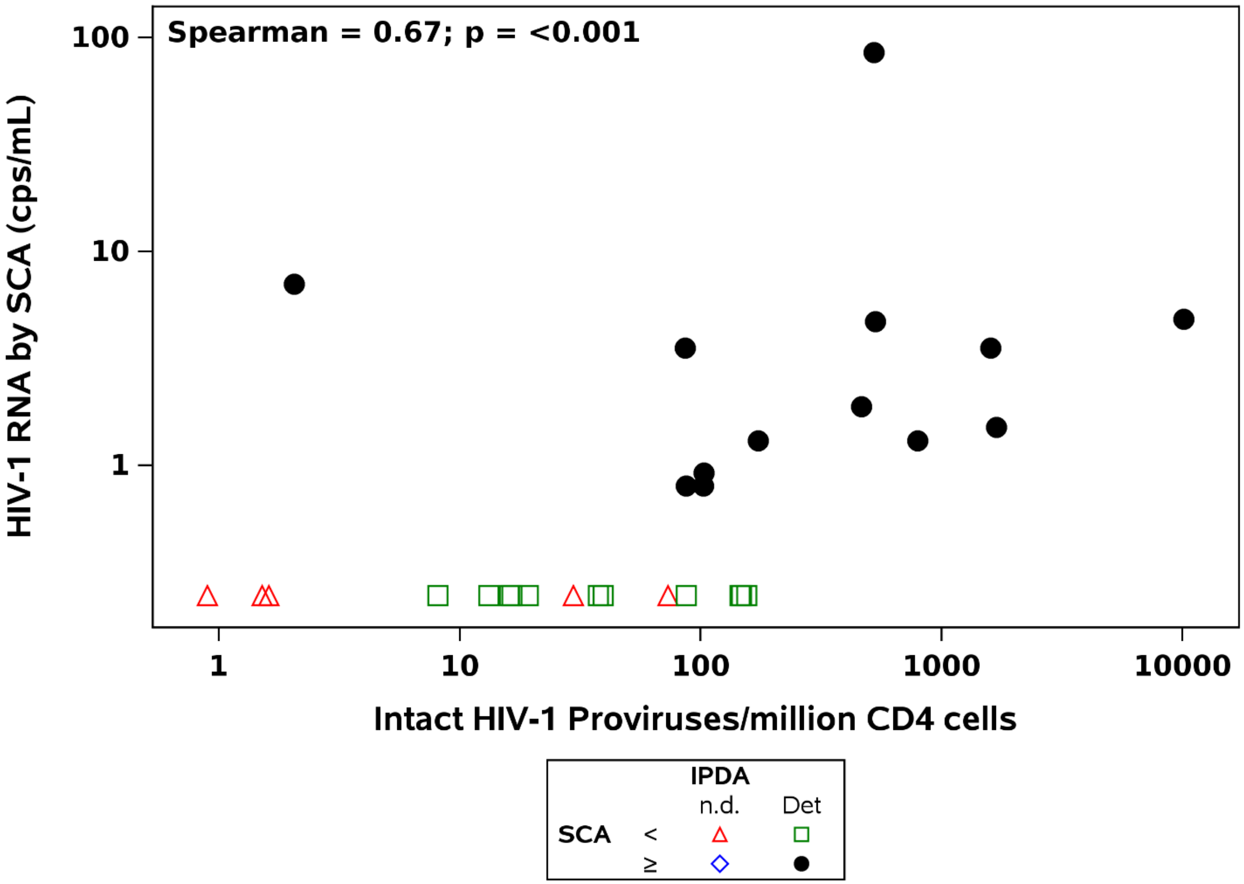

Supplemental Figure 3: SCA vs IPDA at ART year 4 - 6

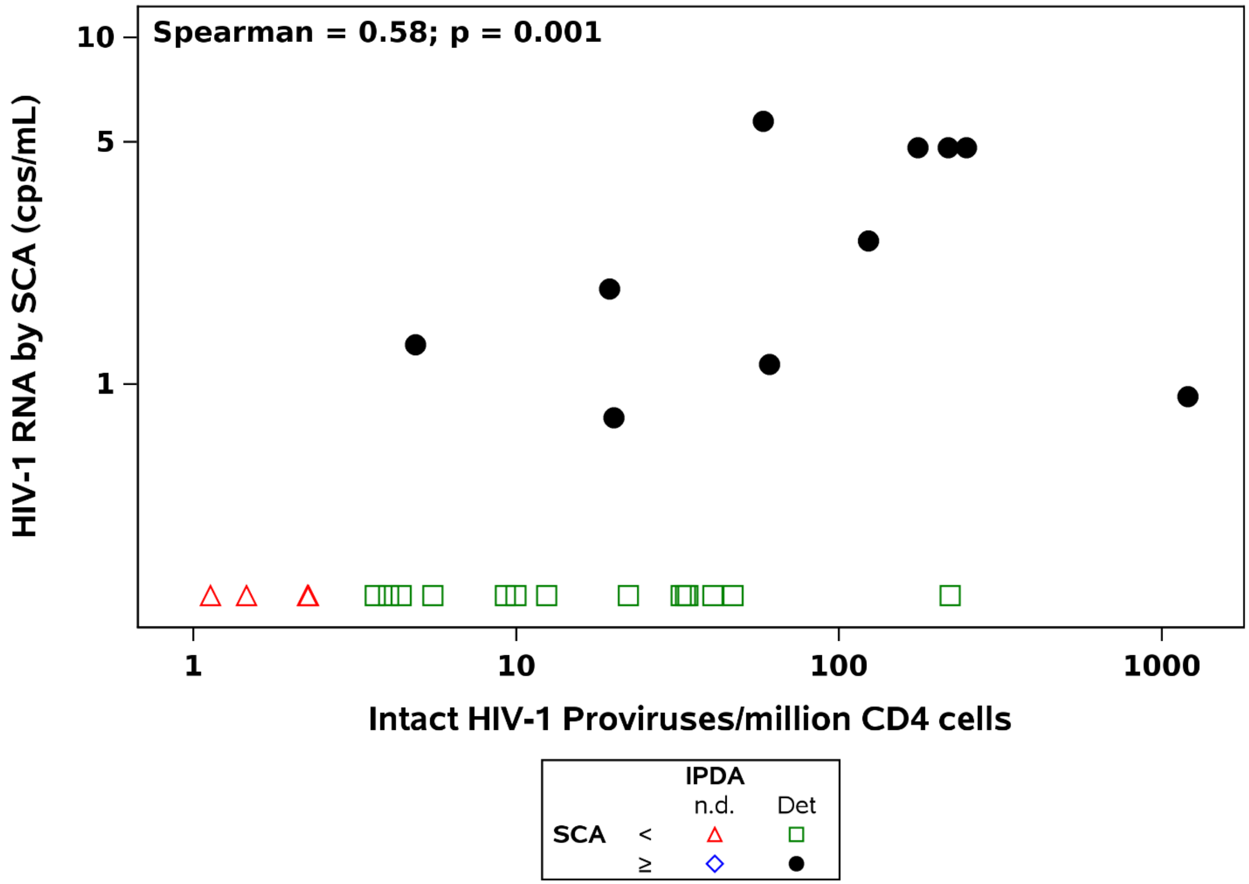

Supplemental Figures 2 & 3:

SCA vs IPDA at ART years 2 and 4 – 6, respectively.

Supplemental Table 2: Participants treated during chronic infection (PWH-CHI): SCA below vs above assay limit, ART yr 2 vs 4 (cps/mL)

		ART Year 2	
		**< 0.49**	**≥ 0.49**
ART Year 4	< 0.49	6 (100%)	2 (25%)
	≥ 0.49	0 (0%)	6 (75%)

Supplemental Table 3: Participants treated during chronic infection (PWH-CHI): SCA below vs above assay limit, ART yr 2 vs 6 (cps/mL)

		ART Year 2	
		**< 0.49**	**≥ 0.49**
ART Year 6	< 0.49	5 (83%)	2 (25%)
	≥ 0.49	1 (17%)	6 (75%)

Supplemental Table 4: Participants treated during chronic infection (PWH-CHI): SCA below vs above assay limit, ART yr 4 vs 6 (cps/mL)

		ART Year 4	
		**< 0.49**	**≥ 0.49**
ART Year 6	< 0.49	6 (75%)	1 (17%)
	≥ 0.49	2 (25%)	5 (83%)
